# Activated Platelets Induce an Anti-Inflammatory Response of Monocytes/Macrophages through Cross-Regulation of PGE_2_ and Cytokines

**DOI:** 10.1155/2017/1463216

**Published:** 2017-05-16

**Authors:** Bona Linke, Yannick Schreiber, Bettina Picard-Willems, Patrick Slattery, Rolf M. Nüsing, Sebastian Harder, Gerd Geisslinger, Klaus Scholich

**Affiliations:** ^1^Department of Clinical Pharmacology, University Hospital Frankfurt, Frankfurt, Germany; ^2^Fraunhofer Institute of Molecular Biology and Applied Ecology-Project Group Translational Medicine and Pharmacology (IME-TMP), Frankfurt, Germany

## Abstract

Platelets are well known for their role in hemostasis and are also increasingly recognized for their roles in the innate immune system during inflammation and their regulation of macrophage activation. Here, we aimed to study the influence of platelets on the production of inflammatory mediators by monocytes and macrophages. Analyzing cocultures of platelets and murine bone marrow-derived macrophages or human monocytes, we found that collagen-activated platelets release high amounts of prostaglandin E_2_ (PGE_2_) that leads to an increased interleukin- (IL-) 10 release and a decreased tumor necrosis factor (TNF) *α* secretion out of the monocytes or macrophages. Platelet PGE_2_ mediated the upregulation of IL-10 in both cell types via the PGE_2_ receptor EP2. Notably, PGE_2_-mediated IL-10 synthesis was also mediated by EP4 in murine macrophages. Inhibition of TNF*α* synthesis via EP2 and EP4, but not EP1, was mediated by IL-10, since blockade of the IL-10 receptor abolished the inhibitory effect of both receptors on TNF*α* release. This platelet-mediated cross-regulation between PGE_2_ and cytokines reveals one mechanism how monocytes and macrophages can attenuate excessive inflammatory responses induced by activated platelets in order to limit inflammatory processes.

## 1. Introduction

Platelets are no longer solely seen as key players in thrombosis and hemostasis. Within the past few years, novel roles of platelets in the fields of wound healing, immune defense, and inflammation have been described [1, 2]. At sites of injuries or infections, platelets are the first cells to be recruited to the vascular endothelium. There they interact with various cell types, including monocytes, neutrophils, and endothelial cells, and, thereby, regulate cellular adhesion and extravasation [3, 4]. Most platelet functions and their interactions with other cell types are restricted to events taking place within the blood vessels. Recently, we found that platelets colocalize with macrophages outside of the blood vessels in several models for cutaneous inflammation and suppress the expression of anti-inflammatory markers and enhance the synthesis of proinflammatory mediators in the associated macrophages [5]. Monocytes in the bloodstream and macrophages in tissues play central roles in various immunological reactions. When activated, these cells synthesize and release a large amount of proinflammatory cytokines and chemokines [6] but are also known to regulate inflammation by producing anti-inflammatory mediators, like IL-10 [7–9]. Platelets are known to induce contrasting answers in macrophages in regard to pro- and anti-inflammatory phenotypes, depending on the underlying pathology, site of inflammation, and experimental model employed [2]. For example, thrombin-activated platelets have been reported to bind to circulating monocytes, thereby inducing the production of proinflammatory cytokines by those cells and thus promoting a proinflammatory phenotype [2, 10] and to increase cytokine and chemokine synthesis in macrophages [11–13]. Depending on the experimental setting, microparticles released from platelets can either enhance proinflammatory effects of macrophages [14, 15] or inhibit proinflammatory cytokine/chemokine secretion [16, 17]. The mechanisms by which platelets and platelet-derived microparticles modulate monocyte or macrophage activity are not fully understood but seem to depend in part on a direct interaction, possibly mediated by adhesion receptors such as CD62P (P-selectin), and in part on the release of soluble factors from platelets [18–20]. Notably, platelets store in granules a wide variety of signaling factors (e.g., mitogenic and angiogenic factors, chemokines (i.e., CXCL4), serotonin, histamine, nucleotides, and proteases), which are released upon platelet activation and have the potential to induce or to modulate proinflammatory responses of macrophages [21]. In addition, platelets are able to synthesize lipid mediators like thromboxane A2 (TXA2) [5, 22, 23] or PGE_2_ [24, 25] upon activation. Furthermore, platelets have been shown to convert human peripheral blood circulating monocytes to IL-10-producing regulatory monocytes [26]. The anti-inflammatory cytokine IL-10, which exerts its effects via binding to the IL-10 receptor (IL-10 R), is known to control the inflammatory process by suppressing the production of proinflammatory cytokines such as tumor necrosis factor alpha (TNF*α*) in monocytes/macrophages [27]. It has been shown that activated platelets enhance IL-10 secretion and reduce TNF*α* secretion by monocytes in order to counteract exaggerated proinflammatory immune responses in vivo [28]. In addition, platelet-rich plasma (PRP) has a strong anti-inflammatory capacity based on the suppression of the synthesis of TNF*α* [29]. In macrophages, the production of IL-10 has been shown to be upregulated by the PGE_2_, another major product of monocytes/macrophages [30], which is also formed and released by platelets [24, 25]. PGE_2_ exerts its effects by four specific cell membrane-associated G protein-coupled receptors, termed EP1, EP2, EP3, and EP4_,_ which differ in their signal transduction pathways [31]. While in bone marrow dendritic cells (BM-DCs), most effects of PGE_2_ are mediated via EP2 and EP4 stimulate the production of IL-10 [32]; the involved receptors in monocytes/macrophages are not known. Thus, platelet-induced intracellular signaling in monocytes or macrophages is highly complex and only incompletely understood. Here, we observed that platelets release PGE_2_ after activation with collagen, leading to an increased release of IL-10 from murine macrophages as well as human monocytes, which in turn suppress TNF*α*. Moreover, we identified the receptors, by which PGE_2_ mediates the upregulation of IL-10 and the suppression of TNF*α* in monocytes/macrophages. In murine macrophages, the EP2 and EP4 receptors were proven to mediate the multi-cytokine-modulating effect of PGE_2_, whereas in human monocytes solely, the EP2 receptors are involved. The TNF*α*-decreasing effect induced by activation of EP1 was demonstrably uncoupled from IL-10 and mediated via a separate pathway.

## 2. Methods

### 2.1. Animals

C57BL/6N mice were supplied by JANVIER LABS (Le Genest-Saint-Isle, France). COX-1-, EP2-, and EP4-deficient mice were described previously [33–35]. In all experiments, the Ethics guidelines of the Public Health Services for investigations in conscious animals were obeyed and the procedures were approved by the local Ethics Committee.

### 2.2. Reagents

Refludan was obtained from Pharmion (Berlin, Germany). Horm collagen was purchased from Takeda Austria GmbH (Linz, Austria). mCSF was obtained from PeproTech (Hamburg, Germany). Mouse IL-10 R*α* antibody was purchased from R&D Systems (Minneapolis, USA). EP receptor-specific ligands (EP2 agonist butaprost (free acid), EP3 agonist sulprostone, and EP4 agonist L-902,688) were purchased from Cayman Chemical (Michigan, USA). EP1 agonist ONO-Di-004 was kindly provided by Dr. Maruyama (ONO Pharmaceuticals, Sekurei, Japan).

### 2.3. Prostanoid Measurement

Analysis of prostanoids was performed using liquid chromatography-electrospray ionization-tandem mass spectrometry (LC-ESI-MS/MS). The LC-MS/MS system consisted of a hybrid triple quadrupole linear ion trap mass spectrometer 5500 QTRAP (SCIEX, Darmstadt, Germany) equipped with a Turbo-V-source operating in negative ESI mode, an Agilent 1200 binary pump, a degasser (Agilent, Waldbronn, Germany), and an HTC Pal autosampler (Chromtech, Idstein, Germany). Data acquisition was done using Analyt V 1.6.2.

For the analysis of prostanoids, the supernatant samples were spiked with the respective isotopically labeled internal standards; mixed with 200 *μ*l PBS, 100 *μ*l EDTA solution (0.15 M), and 600 *μ*l ethyl acetate; and vortexed. The organic phase was removed, and the extraction repeated with 600 *μ*l ethyl acetate. The organic fractions were combined and evaporated at a temperature of 45°C under a gentle stream of nitrogen. The residues were reconstituted with 50 *μ*l of acetonitrile/water/formic acid (20 : 80 : 0.0025, *v*/*v*) in glass vials. The chromatographic separation was carried out using a Synergi Hydro-RP column (150 × 2 mm I.D., 4 *μ*m particle size, and 80 Å pore size from Phenomenex, Aschaffenburg, Germany). A linear gradient was employed at a flow rate of 300 *μ*l/min. The mobile phase A was water/formic acid (100 : 0.0025, *v*/*v*) and mobile phase B was acetonitrile/formic acid (100 : 0.0025, *v*/*v*). The total run time was 16 min and injection volume 20 *μ*l. Retention times of 6-keto-PGF_1*α*_, TXB_2_, PGF_2*α*_, PGE_2_, and PGD_2_ were 7.2 min, 7.8 min, 8.1 min, 8.5 min, and 8.9 min, respectively. The precursor-to-product ion transitions used for quantification were m/z 351.1 → m/z 315.0 for PGE_2_ and PGD_2_, m/z 353.1 → m/z 291.0 for PGF_2*α*_, m/z 369.1 → m/z 162.9 for 6-keto-PGF_1*α*_, and m/z 369.1 → m/z 169.1 for TXB_2_. The dwell time was set at 50 ms for all transitions. Calibration curves were constructed by plotting the corrected analyte areas versus the corresponding nominal analyte concentrations and performing a quadratic regression with 1/x^2^ weighting.

### 2.4. Cytokine Determination in Cell Culture Medium and Cell Lysate

TNF*α* and IL-10 were measured by ELISA kits from R&D Systems (Minneapolis, USA) according to the manufacturer's protocol. For cell lysis, cells were scraped, washed once with 1x PBS, and sonicated in 100 *μ*l assay buffer. After centrifugation (10,000 ×g) for 3 min at RT, cytokine levels were determined in the supernatant.

### 2.5. Preparation of Murine Platelets

Murine blood was diluted in 0.106 mol/l tri-sodium citrate solution (1 : 10) and platelet-rich plasma (PRP) was generated by centrifugation (300 ×g, 5 min, RT). PRP was centrifuged for 30 min at 1300*g* and the pellet was resuspended in HBSS with Ca^2+^ and Mg^2+^ (Life Technologies) containing 10% citrate buffer (38 mM citrate, 88 mM sodium citrate) and modified Tyrode's buffer 1 (137 mM sodium chloride, 2.7 mM potassium chloride, 10 mM Hepes [N-2-hydroxyethylpiperazine-N0-2-ethanesulfonic acid], 0.36 mM sodium dihydrogen phosphate, 5.5 mM dextrose, pH 6.4) supplemented with 1 U/ml refludan. Platelets were resuspended in HBSS/citrate and FCS-free RPMI medium. Collagen I (3 ng/ml) was used for platelet activation.

### 2.6. Preparation of Murine Bone Marrow-Derived Macrophages

Bone marrow-derived macrophages were generated as follows. The femur and tibia of the hind legs from adult mice were extracted from the muscle tissue. Bone ends were cut and bone marrow cells were extracted by centrifugation with 10,000 ×g for 10 seconds. The cells were differentiated in RPMI1640 with L-glutamine (Life Technologies), 10% FCS, 100 U/ml penicillin, and 100 *μ*g/ml streptomycin and 20 ng/ml mCSF (PeproTech, Hamburg, Germany) for 7 days on 24 well-plates (Greiner Bio-One) Prior to their use, the platelets were incubated for 10 min at room temperature in 8 mM citrate and 88 mM sodium citrate in HBSS, with either 3 ng/ml collagen for activation. 400 *μ*l of this medium containing 30 mio/ml platelets was added to the attached macrophages and incubated for 3 hours at 5% CO_2_ and 37°C in the incubator.

### 2.7. Isolation of Human Platelets and Monocytes

Human platelets and monocytes were isolated using buffy coats, blood samples which are already enriched with white blood cells, and platelets. The blood was centrifuged at 120 ×g to obtain PRP (platelet-rich plasma). After the PRP supernatant was transferred into a clean conical centrifugation tube, PRP was supplemented with 111 ml ACD-A. After collecting the platelets by centrifugation at 750 ×g for 10 minutes, the resulting platelet pellet was resuspended in modified Tyrode's buffer 1 (137 mM sodium chloride, 2.7 mM potassium chloride, 10 mM Hepes [N-2-hydroxyethylpiperazine-N0-2-ethanesulfonic acid], 0.36 mM sodium dihydrogen phosphate, 5.5 mM dextrose, pH 6.4) supplemented with 1 U/ml refludan. Platelets were resuspended in a modified Tyrode's buffer 2 (same composition as the modified Tyrode's buffer 1 with additional 2 mM calcium chloride, 2 mM magnesium chloride, and 0.02 U/ml apyrase; pH 7.4).

The remaining blood is layered on to Histopaque-1077 (Sigma-Aldrich) and centrifuged without break at 400 ×g for 30 minutes at room temperature. The upper layer was discarded and the interface containing mononuclear cells was transferred into a clean conical centrifugation tube. The cells were washed by adding 40 ml isotonic phosphate buffer. After centrifugation at 250 ×g for 3 minutes, the supernatant was discarded and remaining erythrocytes were lysed dissolving the cell pellet in 5 ml of erythrocyte lysis buffer (155 mM NH4Cl, 10 mM KHCO3, 0.1 mM Na-EDTA, pH 7.2) for 4 min at RT. Residual cells were collected by centrifugation (250 ×g, 3 min) and resuspended in buffer (phosphate-buffered saline (PBS), pH 7.2, 0.5% bovine serum albumin (BSA), 2 mM EDTA). Human monocytes were isolated as follows. 2 mio/ml of peripheral blood mononuclear cells were plated on 24 well-plates (Greiner Bio-One) in an appropriate amount of prewarmed Monocyte Attachment Medium from PromoCell (Heidelberg, Germany) and incubated for 45 minutes at 5% CO_2_ and 37°C in the incubator. After aspirating the supernatant, the attached monocytes were washed twice with 1x PBS. 400 *μ*l of Monocyte Base Medium from PromoCell, containing 200 mio/ml platelets was added to the attached monocytes and incubated for 3 hours at 5% CO_2_ and 37°C in the incubator. Amounts of prostaglandins were measured using cell culture supernatants and obtained by centrifugation (250 ×g, 3 min).

### 2.8. Statistics

Experiments with two treatment groups were analyzed using Student's *t*-test. Experiments with more than two groups were analyzed using ANOVA followed by post hoc tests. Significance was accepted at *P* < 0.05.

## 3. Results

### 3.1. Collagen-Activated Platelets Selectively Regulate the Synthesis of IL-10 and TNF*α* in Cocultures with Murine Macrophages

**Figure 1:**
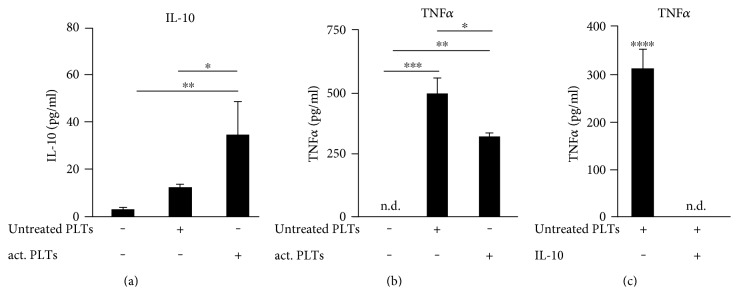
Collagen-activated platelets selectively regulate the synthesis of IL-10 and TNF*α* in cocultures with murine macrophages. (a), (b) Murine bone marrow-derived macrophages (BMDM) were incubated alone, with untreated platelets (untreated PLTs) or with platelets prestimulated with collagen (1 *μ*g/ml; act. PLTs) for 3 hours and the concentrations of IL-10 and TNF*α* were determined by ELISA. (c) Murine BMDM were incubated with untreated platelets with or without murine IL-10 (125 pg/ml). TNF*α* levels were determined in the medium. Data are presented as mean ± SEM from 4 experiments. One-way ANOVA/Bonferroni ^∗^*P* < 0.05, ^∗∗^*P* < 0.01, ^∗∗∗^*P* < 0.002, ^∗∗∗∗^*P* < 0.0004.

Since it has been previously described that thrombin receptor agonist peptide- (TRAP-) activated platelets enhance IL-10 secretion and reduce TNF*α* secretion by monocytes [28], we investigated whether activation with collagen exerts the same effects. Therefore, we incubated murine bone marrow-derived macrophages (BMDM) alone, with untreated platelets, or with collagen-activated platelets and determined the effect of the platelets on the cytokine release. In control experiments, no IL-10 and TNF*α* could be detected in supernatants of untreated as well as collagen-activated platelets (data not shown). The release of IL-10 and TNF*α* was only observed in cocultures with macrophages. We found that collagen-activated platelets induced a 2.82-fold stronger increase of extracellular IL-10 than untreated platelets in cocultures with BMDM (Figure 1(a)). In contrast to IL-10, both untreated and, to a lesser extent, collagen-activated platelets upregulated the secretion of TNF*α* (Figure 1(b)). To show that there is no relevant number of unstimulated platelets which are getting activated during the coincubation with macrophages, we have performed the coincubation with murine macrophages employing untreated as well as BAPTA-AM-inactivated platelets. In both cases, similar amounts of TNF*α* (Figure 1(b)/supplementary data 1 available online at https://doi.org/10.1155/2017/1463216) were seen. To analyze the direct influence of IL-10 on the platelet-induced release of TNF*α*, cocultures of murine BMDM and untreated platelets were incubated with murine IL-10 (125 pg/ml) and the TNF*α* concentration in the medium was determined after 3 hours. IL-10 completely blocked the platelet-induced release of TNF*α* from murine macrophages (Figure 1(c)). Thus, we demonstrated that collagen-activated platelets enhance IL-10 secretion which in turn mediates the reduction of TNF*α*-secretion by murine BMDMs.

### 3.2. Cross-Regulation of PGE_2_ and TNF*α* through an Induction of IL-10 by Platelet-Derived PGE_2_

**Figure 2:**
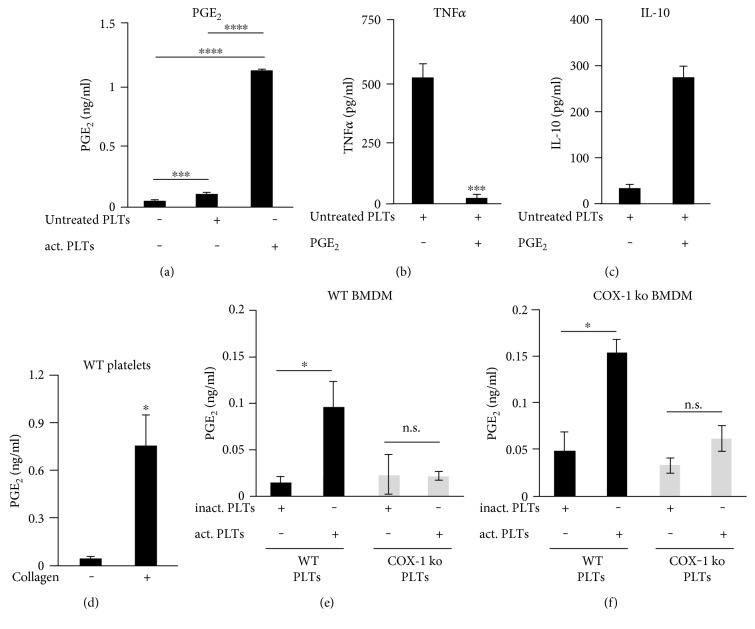
Cross-regulation of PGE_2_ and TNF*α* through an induction of IL-10 in murine macrophages by platelet derived PGE_2_. (a) Murine BMDM were incubated alone, with untreated platelets, or with collagen-activated platelets for 3 hours. (b), (c) Murine BMDM were incubated with untreated platelets with or without PGE_2_ (1 *μ*M) for 3 hours. (d) Murine platelets were incubated with or without collagen (1 *μ*g/ml) for 30 minutes. (e), (f) BMDM from wild-type mice (b) or COX-1-deficient mice (c) were coincubated with BAPTA-AM-inactivated platelets (30 *μ*M) or with collagen-activated platelets isolated from wild-type and COX-1-deficient mice for 3 hours. The concentrations of PGE_2_ in the medium were determined by LC-MS/MS. IL-10 levels and TNF*α* levels in the medium were determined by ELISA. Data are presented as mean ± SEM from 4 experiments. One-way ANOVA/Bonferroni ^∗^*P* < 0.05, ^∗∗^*P* < 0.01, ^∗∗∗^*P* < 0.002, ^∗∗∗∗^*P* < 0.0004.

In macrophages, the production of IL-10 has been shown to be upregulated by the prostaglandin PGE_2_, another major product of monocytes/macrophages [30], which is also formed and released by platelets [24, 25]. In cocultures of platelets and murine BMDMs, we observed that collagen-activated platelets induced a stronger PGE_2_ release than untreated platelets (Figure 2(a)). In order to elucidate a possible cross-regulation of TNF*α* and PGE_2_, we determined whether exogenous PGE_2_ can decrease the release of TNF*α* from platelet-stimulated murine macrophages. Therefore, we coincubated murine BMDM with untreated platelets, causing a strong TNF*α* release (505 ± 55 pg/ml) released from the macrophages (Figure 2(b)). However, coincubation with PGE_2_ (1 *μ*M) completely abolished the platelet-induced TNF*α* secretion. To study whether or not the blockage of the platelet-induced TNF*α* release by PGE_2_ is due to an inhibition of intracellular TNF*α* production, we analyzed the concentrations of TNF*α* in cell lysates of murine BMDMs preincubated with untreated platelets (Supplementary data 2). We found that PGE_2_ nearly completely abrogated the platelet-induced accumulation of intracellular TNF*α* in murine macrophages from 263 ± 55.5 pg/ml down to 33.6 pg/ml.

For certain cell types, a cross-regulation of PGE_2_ and TNF*α* [36, 37] through an induction of IL-10 by PGE_2_ is reported [32, 38]. To investigate whether the observed IL-10 upregulation can be mediated via PGE_2_, we treated a coculture of untreated platelets and BMDMs with exogenous PGE_2_ (1 *μ*M). We found that the application of PGE_2_ leads to a 7.7-fold higher release of IL-10 (Figure 2(c)), implicating an interaction between PGE_2_ and IL-10 signaling, both increasingly released in cocultures of murine macrophages and activated platelets.

Due to the fact that PGE_2_ can be generated and released by platelets [24, 25] as well as monocytes/macrophages [36], we verified the origin of PGE_2_ in our in vitro model system. Therefore, we first determined the amounts of PGE_2_ within supernatants of platelets in response to collagen activation. Collagen treatments caused a significant release of PGE_2_ from the isolated platelets (Figure 2(d)), revealing that activated platelets are indeed capable to provide high amounts of PGE_2_. The generation of PGE_2_ in platelets is catalyzed solely by the cyclooxygenase-1 (COX-1) isoform, whereas within monocytes/macrophages, a second isoform named COX-2 can lead to the production of PGE_2_ during inflammatory settings [39]. Thus, we used COX-1-deficient platelets to identify the origin of extracellular PGE_2_ in the cocultures of platelets and BMDM. We incubated bone marrow-derived macrophages from wild-type mice (Figure 2(e)) or COX-1-deficient mice (Figure 2(f)) with BAPTA-AM-inactivated platelets or with collagen-activated platelets isolated from wild-type and COX-1-deficient mice and determined the concentrations of PGE_2_. In both wild-type and COX-1-BMDM cocultures, only collagen-activated wild-type platelets led to an increased level of PGE_2_, demonstrating that platelets are the source of PGE_2,_ due to the fact that COX-1-deficient platelets are not capable of building any PGE_2_.

In conclusion, these results suggest that PGE_2_ released from collagen-activated platelets leads to the release of IL-10 from murine macrophages which in turn suppresses the intracellular accumulation and the release of TNF*α*.

### 3.3. EP2 and EP4 Receptors Induce IL-10 Release from Murine Macrophages in Response to Activated Platelets

**Figure 3:**
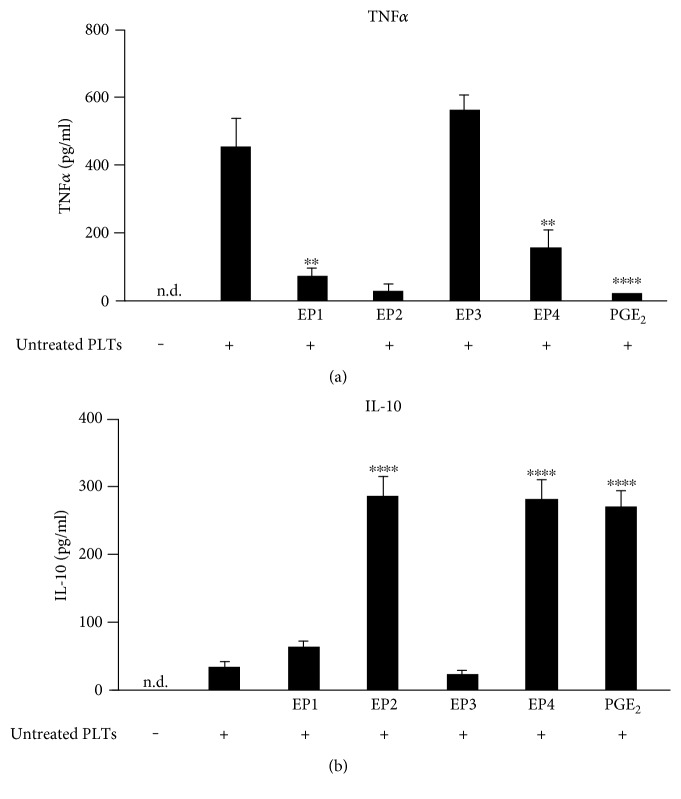
Influence of EP receptor agonists on the release of TNF*α* and IL-10 from platelet-stimulated murine macrophages. Murine BMDM were incubated alone or with untreated platelets and the respective EP agonist concentrations for 3 hours (400 nM ONO-Di-004, 170 nM butaprost, 10 nM sulprostone, 5 nM L-902,688, 1 *μ*M PGE_2_). TNF*α* (a) or IL-10 (b) were determined by ELISA. Data are presented as mean ± SEM from 4 experiments. One-way ANOVA/Bonferroni ^∗^*P* < 0.05, ^∗∗^*P* < 0.01, ^∗∗∗^*P* < 0.002, ^∗∗∗∗^*P* < 0.0004.

The effects of PGE_2_ on monocytes/macrophages are exerted by four subtypes of specific G protein-coupled receptors on their plasma membranes (EP1, EP2, EP3, and EP4) [32]. In order to examine which EP receptor is mediating the suppression of TNF*α* by PGE_2_, we generated cocultures of BMDM and untreated platelets and treated them with the EP agonists for 3 h (Figure 3(a)). For all EP agonists, several different concentrations were tested (data not shown) and concentrations 10 times higher than the EC_50_ value indicated by the provider were used in the following experiments. Treatment of BMDM cultures with untreated platelets increased the TNF*α* concentration in the cell culture medium from undetectable levels to 441 ± 78 pg/ml (*n* = 4) (Figure 3(a)). The EP1 agonist ONO-Di-004 (400 nM) and the EP2 agonist butaprost (170 nM) almost completely inhibited platelet-induced TNF*α* release similar to the actions of PGE_2_. The EP4 agonist L-902,688 (5 nM) showed the same effect but to a lesser extent. In contrast, 10 nM of the EP3 agonist sulprostone (K_i_ 0.6 nM) exhibited no effect on the TNF*α* release, which was not surprising because former reports already described the absence of an effect of sulprostone on IL-10 secretion from bone marrow cells [40, 41]. Thus, PGE_2_ seems to suppress platelet-induced TNF*α* in murine macrophages via its receptors EP1, EP2, and EP4.

To study whether or not these three EP receptors also induce an IL-10 release, we determined the extracellular IL-10 levels from the same cocultivated cells. Unstimulated BMDM exhibited no detectable IL-10 level, whereas the coincubation with untreated platelets only slightly elevated IL-10 in cell supernatants after 3 h up to 34.8 ± 8.3 pg/ml, while PGE_2_ (1 *μ*M) increased the IL-10 production in macrophages to 270 ± 24.5 pg/ml. The EP2 agonist butaprost and the EP4 agonist L-902,688 were nearly as potent as PGE_2_ in increasing IL-10. In contrast, neither the EP1 agonist ONO-Di-004 nor the EP3 agonist sulprostone increased the IL-10 release. Thus, the suppression of TNF*α* by PGE_2_-induced IL-10 release in murine macrophages in response to activated platelets seems to be mediated via EP2 and EP4 receptors, while EP1 activation reduces TNF*α* via a different pathway.

### 3.4. EP2 and EP4 Decrease TNF*α* through IL-10 and Its Receptor IL-10 R

**Figure 4:**
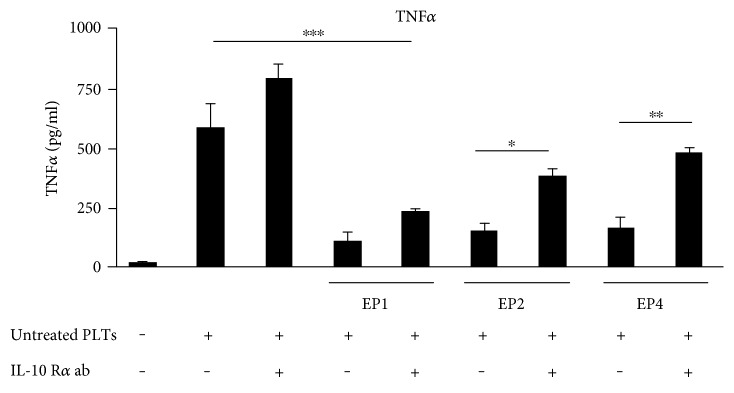
EP2 and EP4 decrease TNF*α* through IL-10 and its receptor IL-10 R. Murine BMDM were incubated alone or with untreated platelets ± IL-10 R*α* antibody (9 *μ*g/ml) 30 minutes before the application of the respective EP agonist concentrations for 3 hours (40 nM ONO-Di-004, 100 nM butaprost, 5 nM L-902,688, 30 nM PGE_2_). TNF*α* was determined by ELISA. Data are presented as mean ± SEM from 4 preparations. One-way ANOVA/Bonferroni ^∗^*P* < 0.05, ^∗∗^*P* < 0.01, ^∗∗∗^*P* < 0.002.

To examine whether or not PGE_2_ is mediating the inhibition of TNF*α* via IL-10 R, we incubated murine BMDM with untreated platelets and the different EP agonists with or without adding the murine IL-10 R*α* antibody (9 *μ*g/ml). As shown before, untreated platelets caused a significant increase in TNF*α* release from murine macrophages after 3 h, while the EP1 agonist ONO-Di-004, the EP2 agonist butaprost, and the EP4 agonist L-902,688 decreased the TNF*α* release (Figure 4). Importantly, the neutralizing antibody IL-10 R*α* could not reverse the decrease in TNF*α* levels caused by the EP1 receptor agonist ONO-Di-004 but the TNF*α*-decreasing effects of butaprost (60.6%) and L-902,688 (65.5%). Thus, the data show that activated platelets release PGE_2_, which increases the synthesis of IL-10 in murine macrophages via EP2 and EP4, and IL-10 then again suppresses TNF*α* synthesis.

### 3.5. Ex Vivo Validation of the Cross-Regulation between Platelet PGE_2_ and Macrophage IL-10 and TNF*α*

**Figure 5:**
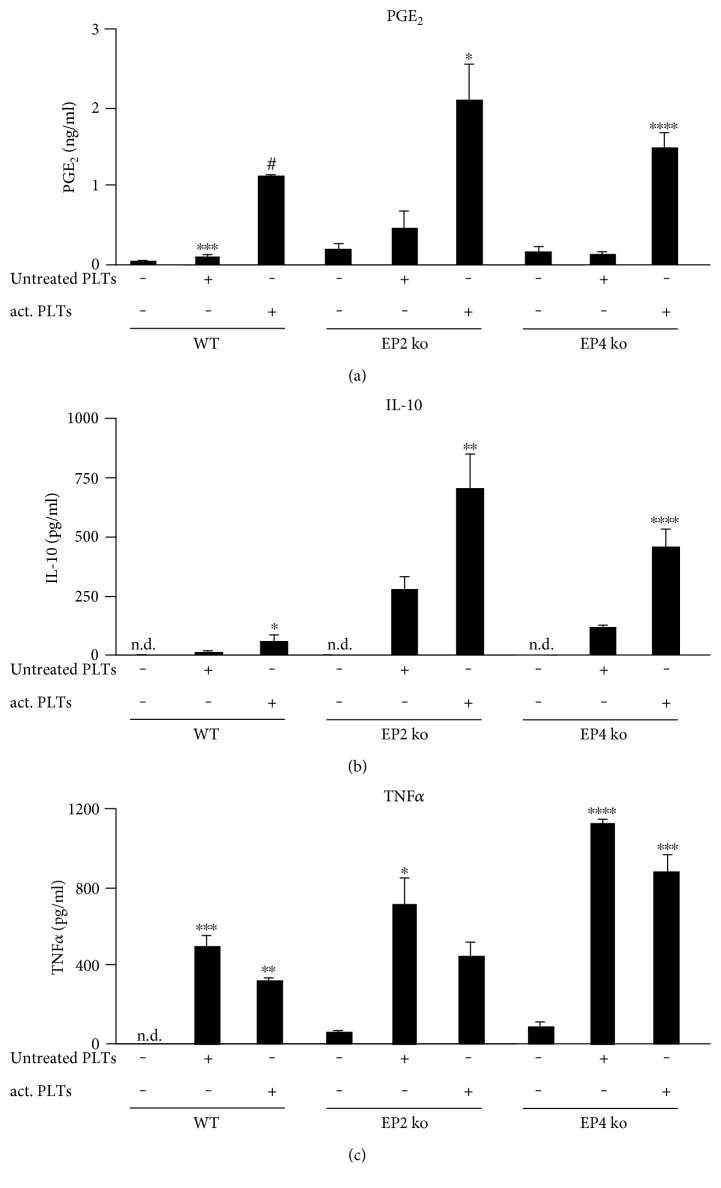
Ex vivo validation of the cross-regulation between platelet PGE_2_ and IL-10 and TNF*α* released from EP2- and EP4-deficient macrophages. Murine BMDM from wild-type, EP2-deficient, and EP4-deficient mice were incubated alone, with untreated platelets (untreated PLTs) or with platelets prestimulated with collagen (1 *μ*g/ml; act. PLTs) for 3 hours. The concentrations of PGE_2_ (a) were determined by LC-MS/MS. IL-10 levels (b) and TNF*α* levels (c) were determined by ELISA. One-way ANOVA/Bonferroni ^∗^*P* < 0.05, ^∗∗^*P* < 0.01, ^∗∗∗^*P* < 0.002, ^∗∗∗∗^*P* < 0.0004, ^#^< 0.00008.

To examine the impact of the absence of EP2 or EP4 on the observed cross-regulation, we determined the amounts of PGE_2_, TNF*α*, and IL-10 in supernatants of BMDM from wild-type, EP2-deficient, and EP4-deficient mice, which were coincubated for 3 hours with untreated or collagen-activated wild-type platelets. In each type of coculture, wild-type platelets released PGE_2_ in response to collagen (Figure 5(a)). Similar to wild-type macrophages, EP2-deficient as well as EP4-deficient macrophages react towards platelet PGE_2_ with an increased release of IL-10 (Figure 5(b)) and a suppression of TNF*α* (Figure 5(c)). In conclusion, the ex vivo data suggest that the genetic deletion of either the EP2 or the EP4 receptor subtypes can be compensated by the other receptor. For that reason, EP2- and EP4-deficient mice are not a suitable model for the analysis of the in vivo impact of the observed cross-regulation.

### 3.6. PGE_2_ Mediates Inhibition of TNF*α* Release from Platelet-Stimulated Human Monocytes

**Figure 6:**
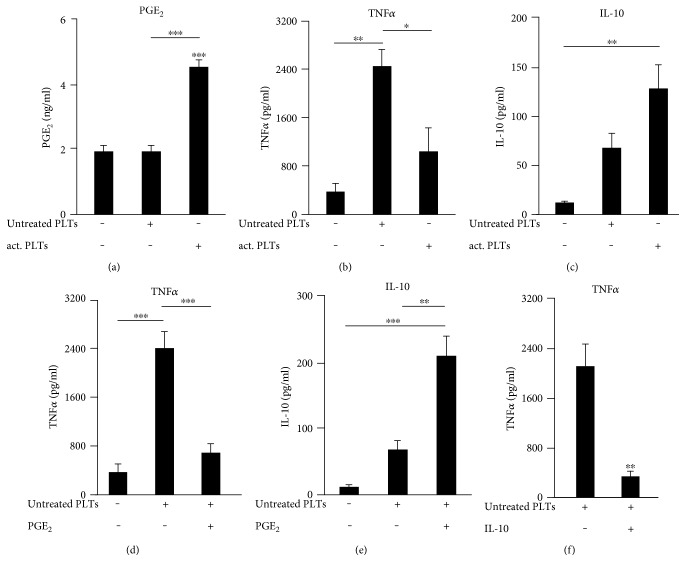
PGE_2_ mediates inhibition of TNF*α* release from platelet-stimulated human monocytes. (a), (b), (c) Human monocytes were incubated alone, with untreated human platelets or with collagen-activated human platelets for 3 hours and PGE_2_ (a), TNF*α* (b), or IL-10 (c) were determined. (d), (e) Human monocytes were incubated alone or with untreated platelets with or without PGE_2_ (1 *μ*M). (f) Human monocytes were incubated with untreated platelets with or without human IL-10 (125 pg/ml). The concentrations of PGE_2_, TNF*α*, and IL-10 within the cell-free supernatants were determined by LC-MS/MS or rather ELISA within cell-free supernatants. Data are presented as mean ± SEM from 4 experiments. One-way ANOVA/Bonferroni (a)–(e) or two-tailed *t*-test (f) ^∗^*P* < 0.05, ^∗∗^*P* < 0.01, ^∗∗∗^*P* < 0.002.

To determine whether human monocytes respond to platelets similar to murine macrophages, we isolated peripheral mononuclear cells out of human blood and let the monocytes attach on cell culture plates. After 3 hours of culture, the supernatants were collected and assayed with PGE_2_, TNF*α*, and IL-10 in the medium. We found that collagen-activated platelets induced a strong PGE_2_ increase in medium (Figure 6(a)), which leads to a decreased TNF*α* release (Figure 6(b)) and a significant upregulation of IL-10 (Figure 6(c)). Thus, human monocytes showed the same correlation between PGE_2_, IL-10, and TNF*α* in response to platelets as observed for murine macrophages. TNF*α* produced by human monocytes in the presence of untreated platelets was significantly decreased by the application of exogenous PGE_2_ (Figure 6(d)) which in turn increased the amount of IL-10 (Figure 6(e)). To analyze the direct influence of IL-10 on the platelet-induced release of TNF*α*, we incubated the coculture of human monocytes and untreated platelets with human IL-10 (125 pg/ml) and determined the concentration of TNF*α* in the supernatant after 3 hours. IL-10 was able to attenuate the platelet-induced release of TNF*α* out of human monocytes (Figure 6(f)). Thus, as seen in murine macrophages, activated platelets release high amounts of PGE_2_ leading to an increased release of IL-10.

### 3.7. EP2 Receptors Induce IL-10 Release from Human Monocytes in Response to Activated Platelets

**Figure 7:**
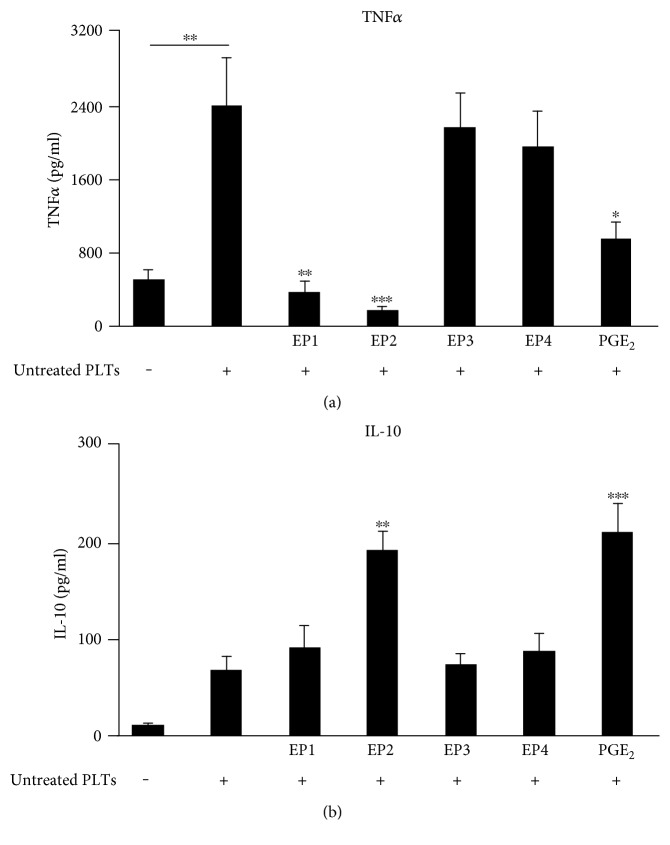
Influence of EP receptor agonists on the release of TNF*α* and IL-10 from platelet-stimulated human monocytes. Human monocytes were incubated alone or with untreated platelets and the respective EP agonist concentrations for 3 hours (400 nM ONO-Di-004, 170 nM butaprost, 10 nM sulprostone, 5 nM L-902,688, 1 *μ*M PGE_2_). TNF*α* (a) or IL-10 (b) were determined by ELISA. Data are presented as mean ± SEM from 4 experiments. One-way ANOVA/Bonferroni ^∗^*P* < 0.05, ^∗∗^*P* < 0.01, ^∗∗∗^*P* < 0.002.

To investigate which EP receptor mediates IL-10 release and inhibition of TNF*α* release, human monocytes were incubated with platelets in absence and presence of EP receptor agonists. EP1 receptor agonist ONO-Di-004 (400 nM) and EP2 agonist butaprost (170 nM) completely blocked the platelet-induced TNF*α* release (Figure 7(a)). As observed for murine macrophages, the EP3 receptor agonist sulprostone (10 nM) exhibited no inhibitory effect on platelet-mediated TNF*α* release from human monocytes. In contrast to murine macrophages, the EP4 agonist L-902,688 (5 nM) did not affect the release of TNF*α*. Thus, PGE_2_ seems to inhibit platelet-induced TNF*α* release out of human monocytes via its receptors EP1 and EP2.

To investigate which of the EP receptors mediates the suppression of TNF*α* by PGE_2_-induced IL-10, we determined the extracellular IL-10. Application of exogenous PGE_2_ (1 *μ*M) led to a 3.1-fold increase of IL-10 production in monocytes up to 212 ± 29 pg/ml and only the EP2 agonist butaprost increased the IL-10 release (Figure 7(b)). In contrast, neither the EP1 receptor agonist ONO-Di-004, the EP3 receptor agonist sulprostone, nor the EP4 receptor agonist L-902,688 affected the IL-10 release from human monocytes. Thus, in human monocytes, EP2 receptors mediate the suppression of TNF*α* by PGE_2_-induced IL-10 in response to activated platelets, causing an anti-inflammatory reaction of monocytes towards activated platelets.

### 3.8. EP2 Decreases TNF*α* through IL-10 and Its Receptor IL-10 R

**Figure 8:**
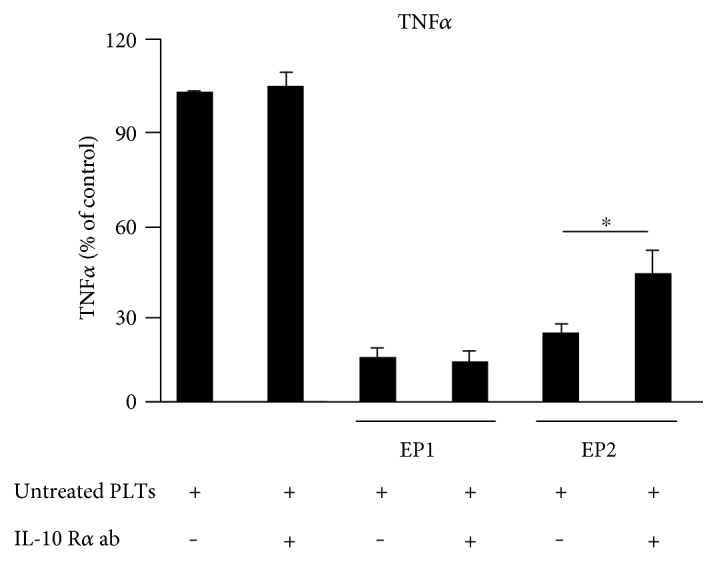
EP2 decreases TNF*α* through IL-10 and its receptor IL-10 R. Human monocytes were incubated with untreated platelets ± IL-10 R*α* antibody (22.5 *μ*g/ml) 30 minutes before the application of the respective EP agonist concentrations for 3 hours (40 nM ONO-Di-004, 75 nM butaprost). TNF*α* was determined by ELISA. Data are presented as mean ± SEM from 4 preparations. One-way ANOVA/Bonferroni ^∗^*P* < 0.05.

To examine whether or not the EP2 agonist butaprost is mediating the inhibition of TNF*α* via IL-10 and its receptor IL-10 R, we incubated cocultures of human monocytes and untreated platelets with the different EP agonists with or without adding the human IL-10 R*α* antibody (22.5 *μ*g/ml). The EP1 agonist ONO-Di-004 and the EP2 agonist butaprost decreased the TNF*α* release from human monocytes significantly (Figure 8). Importantly, the neutralizing antibody IL-10 R*α* increased the TNF*α* levels only in combination with butaprost but not with the EP1 agonist. Thus, IL-10 and its receptor IL-10 R are involved in the suppression of TNF*α* by EP2, while EP1 activation reduces TNF*α* via a different pathway.

## 4. Discussion

Platelets interact with macrophages modulating their inflammatory functions in several inflammatory settings, such as arteriosclerosis and rheumatoid arthritis. Most platelet functions and their interactions with other cell types are restricted to events taking place within the blood vessels. Recently, we found that platelets colocalize with macrophages outside of the blood vessels in several models for cutaneous inflammation and suppress the expression of anti-inflammatory markers and enhance the synthesis of proinflammatory mediators in the associated macrophages (5). However, platelet-induced intracellular signaling in monocytes or macrophages is highly complex and still not completely understood. Our study helps to clarify how activated platelets modulate the production of inflammatory mediators by human monocytes and murine macrophages in early stages of inflammation. We observed that activated platelets selectively release PGE_2_ in cocultures with murine macrophages and human monocytes, which leads to an induction of IL-10 that in turn suppresses the intracellular accumulation as well as the release of TNF*α*. To our knowledge, these data are the first revealing that platelets are inducing an anti-inflammatory reaction of monocytes/macrophages via a cross-regulation of PGE_2_ and TNF*α* through IL-10.

It is not surprising that platelets influence the production of pro- as well as anti-inflammatory mediators in monocytes/macrophages, since it is known that activated platelets upregulate the production of inflammatory cytokines by human peripheral blood mononuclear cells [25] and human monocyte-derived macrophages [42]. For example, platelet-monocyte interactions promote the release of proinflammatory IL-8, TNF*α*, and IL-6 upon coincubation in vitro [12, 42]. On the other hand, platelets have been shown to dampen inflammatory responses by triggering IL-10 and downregulating IL-6 and TNF*α* release by monocytes [2] as well as macrophages [43]. From the literature, it is known that TRAP-activated platelets enhance IL-10 secretion and reduce TNF*α* secretion by monocytes (19). In our study, we observed three major modulatory effects induced by collagen-activated platelets as follows: (i) activated platelets led to an enhanced release of PGE_2_, (ii) an attenuation of the synthesis as well as the release of TNF*α*, and (iii) an upregulated induction of IL-10. Whereas the actions of TNF*α* as a proinflammatory mediator and IL-10 as an anti-inflammatory mediator are clearly defined, PGE_2_ fulfills contrasting roles in the induction of inflammatory responses. Its well-known proinflammatory properties are the reason for the clinical use of the anti-inflammatory-acting COX inhibitors [44–46], while several functions of monocytes/macrophages are not proinflammatory but rather anti-inflammatory [47]. In this regard, PGE_2_ inhibits the induction of proinflammatory cytokines in monocytes/macrophages [48] and can upregulate anti-inflammatory mediators, like IL-10 in case of macrophages [30].

In the experimental model we employed, PGE_2_ seems to exert anti-inflammatory effects. The release of PGE_2_ from activated platelets in cocultures with monocytes/macrophages led to an induction of IL-10 that in turn suppressed the intracellular accumulation as well as the release of TNF*α*. Due to the fact that uncontrolled expression of TNF*α* would lead to serious consequences, culminating in multiorgan failure and death [11], we suggested that the observed cytokine-modulating feature of PGE_2_ is contributing to the resolution of the inflammation in order to prevent serious tissue injury.

**Figure 9:**
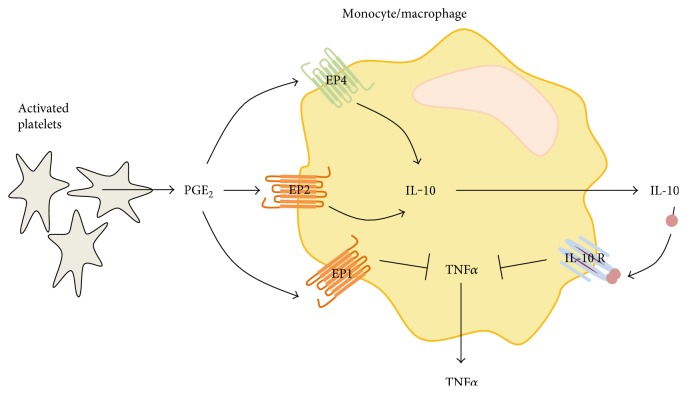
Summary of the platelet-induced crosstalk between PGE_2_, IL-10, and TNF*α* in murine macrophages and human monocytes. Activated platelets produce and release PGE_2_ in cocultures with macrophages. PGE_2_ binds to EP1, EP2 (orange), and in case of murine macrophages additionally to EP4 (green). The binding of PGE_2_ to EP1 decreased synthesis and secretion of TNF*α* independently of IL-10. The activation of EP2 and EP4 in murine macrophages and EP2 in human monocytes increased the secretion of IL-10 which binds to its receptor IL-10 R, leading to an inhibition of the production and release of TNF*α*.

Moreover, we were able to identify the receptors, by which PGE_2_ mediates the upregulation of IL-10 and the suppression of TNF*α* in monocytes/macrophages. In murine macrophages, the EP2 and EP4 receptors mediate the cytokine-modulating effect of PGE_2_. These findings are in accordance with the current literature which reveals that most of the PGE_2_ actions in monocytes/macrophages are mediated via these two receptor subtypes. An in vitro study using the monocytic cell line THP-1 revealed that PGE_2_ regulates cytokine signaling via increased intracellular cAMP levels, mediated by EP2 and/or EP4 receptors [49]. Within bone marrow dendritic cells (BM-DCs), PGE_2_-EP2 and EP4 signaling stimulate the production of IL-10 [32] and inhibited the release and intracellular accumulation of TNF*α* [37]. Our data clearly revealed a difference between monocytes and macrophages according to the receptors involved in mediating the inhibitory effect of PGE_2_. Whereas PGE_2_ mediates the upregulation of IL-10 in both cell types via the EP2 receptors, the EP4 receptors are additionally involved only in case of murine macrophages (Figure 9). Unfortunately, ex vivo analysis using EP2- and EP4-deficient bone marrow-derived macrophages revealed that the genetic deletion of one of these receptor subtypes can be compensated by each other, and thus, the EP2- or the EP4-deficient macrophages can react to platelet PGE_2_ similar than wild-type macrophages with an increased IL-10 release which mediates the suppression of TNF*α*. For that reason, EP2- and EP4-deficient mice are not suitable models for the determination of in vivo effects of the PGE_2_-mediated cross-regulation.

Taken together, the interplay between platelets and monocytes/macrophages during inflammation led to a PGE_2_-induced IL-10 production, which in turn suppresses TNF*α* via EP2 and/or EP4 in order to prevent serious tissue injury. This puts specific EP2 or EP4 agonists in perspective for the anti-inflammatory therapy in inflammatory diseases. Currently, the improvement of clinical symptoms of chronic inflammatory and autoimmune diseases such as rheumatoid arthritis (RA), inflammatory bowel disease (IBD), and psoriasis is reached by the repeated administration of TNF*α*-sequestering antibodies, like the monoclonal antibodies infliximab, adalimumab, and the fusion protein etanercept [22], but therapeutic application is hampered by the lack of oral availability, immunogenic response, restricted distribution, and high costs. PGE_2_ itself is not a therapeutic agent because of its broad biological activities resulting from the activation of all four EP receptors. Compounds that interact specifically with the EP2 receptor may allow a wider, more practical exploitation in the therapy of inflammatory diseases and may additionally act immunosuppressive [50]. Thus, the multi-cytokine-modulating feature of a specific EP2 agonist might be advantageous over the conventional clinical approach represented by the existing anti-TNF*α* therapy.

## Supplementary Material

Supplementary data 1: Collagen-activated platelets selectively regulate the synthesis of TNFα in cocultures with murine macrophages. BMDM from wildtype-mice were co-incubated with BAPTA-AMinactivated platelets (30 μM) or with collagen-activated platelets isolated from wildtype-mice for 3 hours. The concentrations of TNFα in the medium were determined by ELISA. Data are presented as mean ± S.E.M. from 4 experiments. One way ANOVA/Bonferroni ∗P<0.05, ∗∗P<0.01, ∗∗∗P<0.002. Supplementary data 2: Cross-regulation of PGE2 and intracellular TNFα in murine macrophages. Murine BMDM were incubated with untreated platelets with or without PGE2 (1 μM) for 3 hours. Intracellular TNFα levels were determined by ELISA. Data are presented as mean ± S.E.M. from 4 experiments. One way ANOVA/Bonferroni ∗P<0.05, ∗∗P<0.01.

## Abbreviations

BMDM: Bone marrow-derived macrophages
ELISA: Enzyme-linked immunosorbent assay
IL: Interleukin
LC-MS/MS: Liquid chromatography-tandem mass spectrometry
PGE_2_: Prostaglandin E_2_
PLTs: Platelets
TNF*α*: Tumor necrosis factor alpha.

## References

[1] Koenen R. R. (2016). The prowess of platelets in immunity and inflammation. *Thrombosis and Haemostasis*.

[2] Kral J. B., Schrottmaier W. C., Salzmann M., Assinger A. (2016). Platelet interaction with innate immune cells. *Transfusion Medicine and Hemotherapy: Offizielles Organ der Deutschen Gesellschaft für Transfusionsmedizin und Immunhamatologie*.

[3] Zarbock A., Singbartl K., Ley K. (2006). Complete reversal of acid-induced acute lung injury by blocking of platelet-neutrophil aggregation. *Journal of Clinical Investigation*.

[4] Suo J., Linke B., Meyer Dos Santos S. (2014). Neutrophils mediate edema formation but not mechanical allodynia during zymosan-induced inflammation. *Journal of Leukocyte Biology*.

[5] Pierre S., Linke B., Jing S. (2017). GPVI and thromboxane receptor on platelets promote proinflammatory macrophage phenotypes during cutaneous inflammation. *Journal of Investigative Dermatology*.

[6] Mantovani A., Garlanda C. (2013). Platelet-macrophage partnership in innate immunity and inflammation. *Nature Immunology*.

[7] Fiorentino D. F., Vieira P., Mosmann T. R., Howard M., Moore K. W., O'Garra A. (1991). IL-10 acts on the antigen-presenting cell to inhibit cytokine production by Th1 cells. *Journal of Immunology*.

[8] Blanco P., Palucka A. K., Pascual V., Banchereau J. (2008). Dendritic cells and cytokines in human inflammatory and autoimmune diseases. *Cytokine Growth Factor Review*.

[9] Chomarat P., Rissoan M. C., Banchereau J., Miossec P. (1993). Interferon gamma inhibits interleukin 10 production by monocytes. *Journal of Experimental Medicine*.

[10] Passacquale G., Vamadevan P., Pereira L., Hamid C., Corrigall V., Ferro A. (2011). Monocyte-platelet interaction induces a pro-inflammatory phenotype in circulating monocytes. *PloS One*.

[11] Neumann F. J., Gawaz M., Brand K. (1997). Induction of cytokine expression in leukocytes by binding of thrombin-stimulated platelets. *Circulation*.

[12] Weyrich A. S., Elstad M. R., McEver R. P. (1996). Activated platelets signal chemokine synthesis by human monocytes. *Journal of Clinical Investigation*.

[13] Weyrich A. S., McEver R. P., Prescott S. M., Zimmerman G. A. (1995). Monocyte tethering by P-selectin regulates monocyte chemotactic protein-1 and tumor necrosis factor-alpha secretion. Signal integration and NF-kappa B translocation. *Journal of Clinical Investigation*.

[14] Boilard E. N., Larabee P. A., Watts K. (2010). Platelets amplify inflammation in arthritis via collagen-dependent microparticle production. *Science*.

[15] Vasina E. M., Cauwenberghs S., Feijge M. A., Heemskerk J. W., Weber C., Koenen R. R. (2011). Microparticles from apoptotic platelets promote resident macrophage differentiation. *Cell Death Discovery*.

[16] Sadallah S., Eken C., Martin P. J., Schifferli J. A. (2011). Microparticles (ectosomes) shed by stored human platelets downregulate macrophages and modify the development of dendritic cells. *Journal of Immunology*.

[17] Laffont B., Rousseau M., Duchez A. C., Lee C. H., Boilard E., Provost P. (2016). Platelet microparticles reprogram macrophage gene expression and function. *Thrombosis and Haemostasis*.

[18] Soga F., Katoh N., Inoue T., Kishimoto S. (2007). Serotonin activates human monocytes and prevents apoptosis. *The Journal of Investigative Dermatology*.

[19] Stephen J., Emerson B., Fox K. A., Dransfield I. (2013). The uncoupling of monocyte-platelet interactions from the induction of proinflammatory signaling in monocytes. *Journal of Immunology*.

[20] Pervushina O. (2004). Platelet factor 4/CXCL4 induces phagocytosis and the generation of reactive oxygen metabolites in mononuclear phagocytes independently of Gi protein activation or intracellular calcium transients. *Journal of Immunology*.

[21] Gleissner C. A., Shaked I., Little K. M., Ley K. (2010). CXC chemokine ligand 4 induces a unique transcriptome in monocyte-derived macrophages. *Journal of Immunology*.

[22] Dütting S., Bender M., Nieswandt B. (2012). Platelet GPVI: a target for antithrombotic therapy?!. *Trends in Pharmacological Science*.

[23] Jenne C. N., Urrutia R., Kubes P. (2013). Platelets: bridging hemostasis, inflammation, and immunity. *International Journal of Laboratory Hematology*.

[24] Hubertus K., Mischnik M., Timmer J. (2014). Reciprocal regulation of human platelet function by endogenous prostanoids and through multiple prostanoid receptors. *European Journal of Pharmacology*.

[25] Waehre T., Damas J. K., Yndestad A. (2004). Effect of activated platelets on expression of cytokines in peripheral blood mononuclear cells - potential role of prostaglandin E2. *Thrombosis and Haemostasis*.

[26] Inui M., Tazawa K., Kishi Y., Takai T. (2015). Platelets convert peripheral blood circulating monocytes to regulatory cells via immunoglobulin G and activating-type Fcgamma receptors. *BMC Immunology*.

[27] van der Poll T., Jansen J., Levi M., ten Cate H., ten Cate J. W., van Deventer S. J. (1994). Regulation of interleukin 10 release by tumor necrosis factor in humans and chimpanzees. *Journal of Experimental Medicine*.

[28] Gudbrandsdottir S., Hasselbalch H. C., Nielsen C. H. (2013). Activated platelets enhance IL-10 secretion and reduce TNF-alpha secretion by monocytes. *Journal of Immunology*.

[29] Day Y. J., Chen K. H., Huang T. H. (2016). Preactivated and disaggregated shape-changed platelets protected against acute respiratory distress syndrome complicated by sepsis through inflammation suppression. *Shock*.

[30] Strassmann G., Patil-Koota V., Finkelman F., Fong M., Kambayashi T. (1994). Evidence for the involvement of interleukin 10 in the differential deactivation of murine peritoneal macrophages by prostaglandin E2. *Journal of Experimental Medicine*.

[31] Saha A., Biswas A., Srivastav S., Mukherjee M., Das P. K., Ukil A. (2014). Prostaglandin E2 negatively regulates the production of inflammatory cytokines/chemokines and IL-17 in visceral leishmaniasis. *Journal of Immunology*.

[32] Harizi H. (2006). Pivotal role of PGE2 and IL-10 in the cross-regulation of dendritic cell-derived inflammatory mediators. *Cellular and Molecular Immunology*.

[33] Hizaki H., Segi E., Sugimoto Y. (1999). Abortive expansion of the cumulus and impaired fertility in mice lacking the prostaglandin E receptor subtype EP(2). *Proceedings of National Academy of Science U S A*.

[34] Langenbach R., Morham S. G., Tiano H. F. (1995). Prostaglandin synthase 1 gene disruption in mice reduces arachidonic acid-induced inflammation and indomethacin-induced gastric ulceration. *Cell*.

[35] Yoshida K., Oida H., Kobayashi T. (2002). Stimulation of bone formation and prevention of bone loss by prostaglandin E EP4 receptor activation. *Proceedings of National Academy of Science U S A*.

[36] Niho Y., Niiro H., Tanaka Y., Nakashima H., Otsuka T. (1998). Role of IL-10 in the crossregulation of prostaglandins and cytokines in monocytes. *Acta Haematology*.

[37] Vassiliou E., Jing H., Ganea D. (2003). Prostaglandin E2 inhibits TNF production in murine bone marrow-derived dendritic cells. *Cellular Immunology*.

[38] Harizi H., Norbert G. (2004). Inhibition of IL-6, TNF-alpha, and cyclooxygenase-2 protein expression by prostaglandin E2-induced IL-10 in bone marrow-derived dendritic cells. *Cellular Immunology*.

[39] Funk C. D., Kennedy M. E. (1991). Human platelet/erythroleukemia cell prostaglandin G/H synthase: cDNA cloning, expression, and gene chromosomal assignment. *FASEB Journal*.

[40] Józefowski S., Bobek M., Marcinkiewicz J. (2003). Exogenous but not endogenous prostanoids regulate cytokine secretion from murine bone marrow dendritic cells: EP2, DP, and IP but not EP1, EP3, and FP prostanoid receptors are involved. *International Immunopharmacology*.

[41] Harizi H., Grosset C., Gualde N. (2003). Prostaglandin E2 modulates dendritic cell function via EP2 and EP4 receptor subtypes. *Journal of Leukocyte Biology*.

[42] Scull C. M., Hays W. D., Fischer T. H. (2010). Macrophage pro-inflammatory cytokine secretion is enhanced following interaction with autologous platelets. *Journal of Inflammation*.

[43] Ando Y., Oku T., Tsuji T. (2016). Platelets attenuate production of cytokines and nitric oxide by macrophages in response to bacterial endotoxin. *Platelets*.

[44] FitzGerald G. A. (2001). The coxibs, selective inhibitors of cyclooxygenase-2. *New England Journal of Medicine*.

[45] FitzGerald G. A. (2004). Coxibs and cardiovascular disease. *New England Journal of Medicine*.

[46] Scholich K., Geisslinger G. (2006). Is mPGES-1 a promising target for pain therapy?. *Trends in Pharmacological Science*.

[47] Kammer G. M. (1988). The adenylate cyclase-cAMP-protein kinase A pathway and regulation of the immune response. *Immunology Today*.

[48] Zhong W. W., Burke P. A., Drotar M. E., Chavali S. R., Forse R. A. (1995). Effects of prostaglandin E2, cholera toxin and 8-bromo-cyclic AMP on lipopolysaccharide-induced gene expression of cytokines in human macrophages. *Immunology*.

[49] Cheon H., Rho Y. H., Choi S. J. (2006). Prostaglandin E2 augments IL-10 signaling and function. *The Journal of Immunology*.

[50] Fujimoto Y., Ozaki M., Ogino T. (2005). Involvement of prostaglandin receptors (EPR2-4) in in vivo immunosuppression of PGE2 in rat skin transplant model. *International Immunopharmacology*.

