# Supplementary material for: Activated Platelets Induce an Anti-Inflammatory Response of Monocytes/Macrophages through Cross-Regulation of PGE2 and Cytokines
Source: Mediators Inflamm. 2017 May 16;2017:1463216. doi: 10.1155/2017/1463216 (PMC5448075; doi:10.1155/2017/1463216)
Supplement: Supplementary file 1 — Supplementary data 1: Collagen-activated platelets selectively regulate the synthesis of TNFα in cocultures with murine macrophages. BMDM from wildtype-mice were co-incubated with BAPTA-AMinactivated platelets (30 μM) or with collagen-activated platelets isolated from wildtype-mice for 3 hours. The concentrations of TNFα in the medium were determined by ELISA. Data are presented as mean ± S.E.M. from 4 experiments. One way ANOVA/Bonferroni ∗P<0.05, ∗∗P<0.01, ∗∗∗P<0.002. Supplementary data 2: Cross-regulation of PGE2 and intracellular TNFα in murine macrophages. Murine BMDM were incubated with untreated platelets with or without PGE2 (1 μM) for 3 hours. Intracellular TNFα levels were determined by ELISA. Data are presented as mean ± S.E.M. from 4 experiments. One way ANOVA/Bonferroni ∗P<0.05, ∗∗P<0.01. [file 1463216.f1.pptx]

## Slide 1
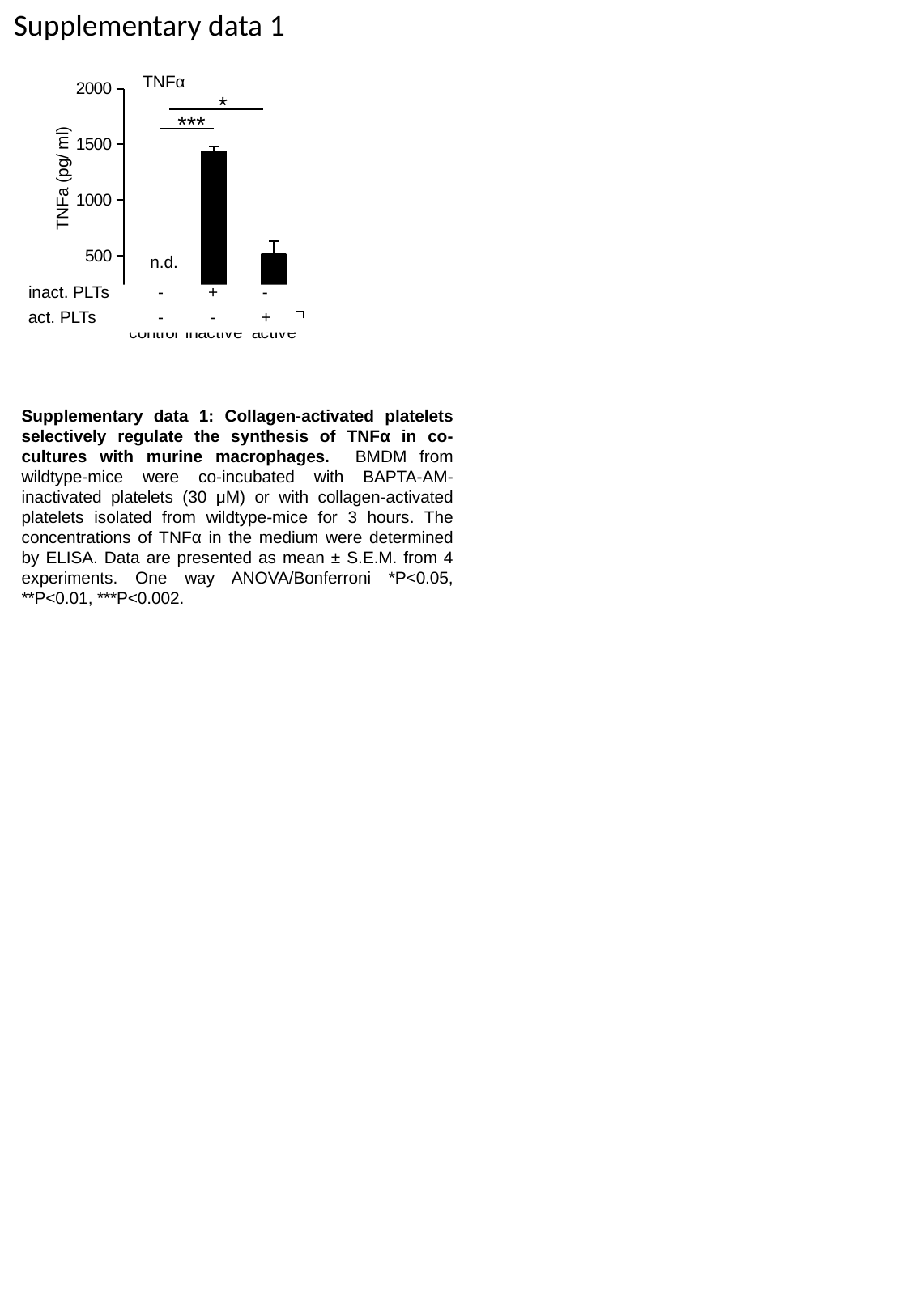

Supplementary data 1
TNFα
### Chart
| Category | |
|---|---|
| control | 0.0 |
| inactive | 1436.7987150000001 |
| active | 509.6522616666666 |*
***
TNFa (pg/ ml)
n.d.
inact. PLTs
-
+
-
act. PLTs
-
-
+
Supplementary data 1: Collagen-activated platelets selectively regulate the synthesis of TNFα in co-cultures with murine macrophages. BMDM from wildtype-mice were co-incubated with BAPTA-AM-inactivated platelets (30 μM) or with collagen-activated platelets isolated from wildtype-mice for 3 hours. The concentrations of TNFα in the medium were determined by ELISA. Data are presented as mean ± S.E.M. from 4 experiments. One way ANOVA/Bonferroni *P<0.05, **P<0.01, ***P<0.002.
