# Supplementary material for: Activated Platelets Induce an Anti-Inflammatory Response of Monocytes/Macrophages through Cross-Regulation of PGE2 and Cytokines
Source: Mediators Inflamm. 2017 May 16;2017:1463216. doi: 10.1155/2017/1463216 (PMC5448075; doi:10.1155/2017/1463216)
Supplement: Supplementary file 2 [file 1463216.f2.pptx]

## Slide 1
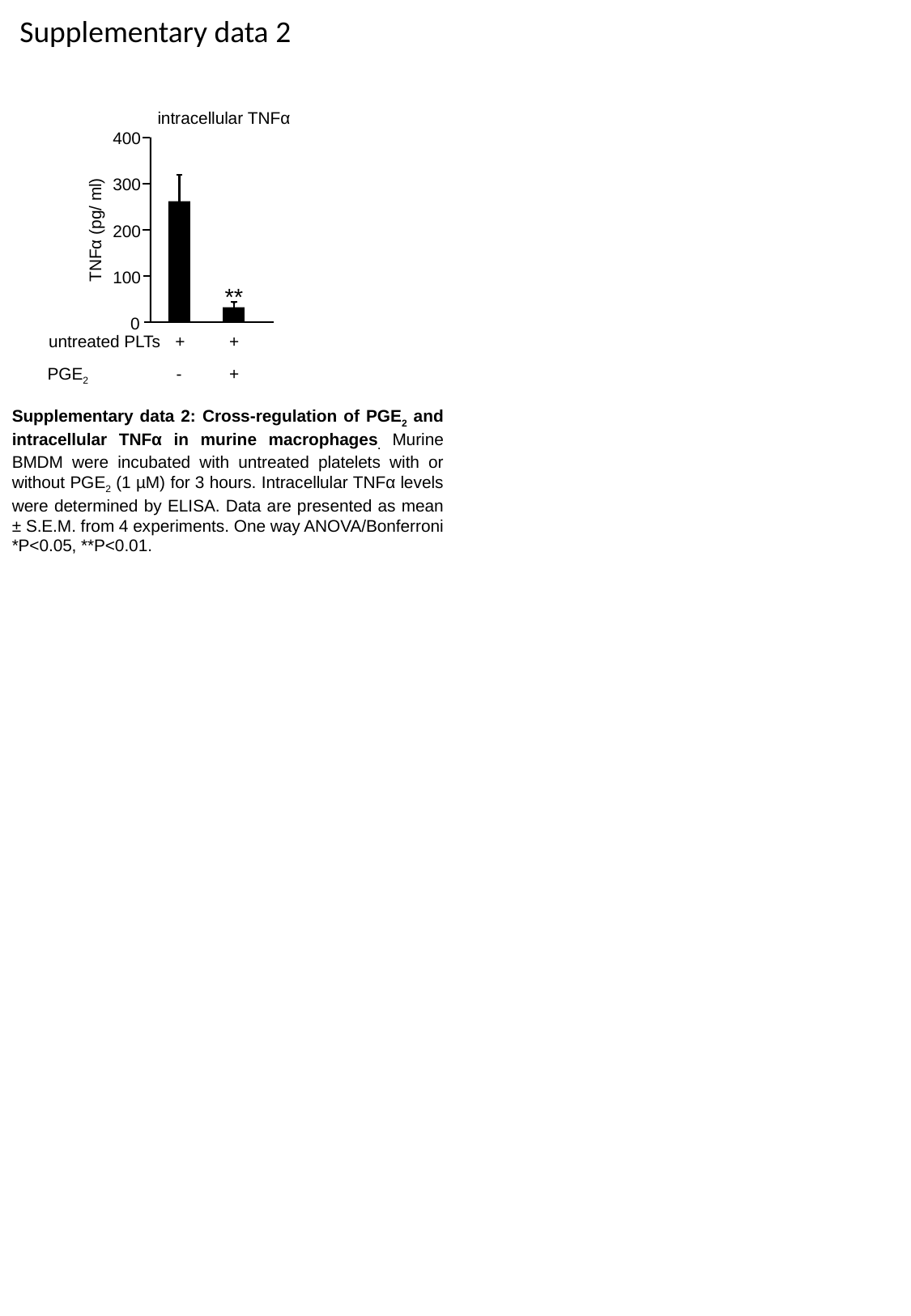

Supplementary data 2
intracellular TNFα
400
300
TNFα (pg/ ml)
200
100
 **
0
untreated PLTs
+
+
PGE2
-
+
Supplementary data 2: Cross-regulation of PGE2 and intracellular TNFα in murine macrophages. Murine BMDM were incubated with untreated platelets with or without PGE2 (1 µM) for 3 hours. Intracellular TNFα levels were determined by ELISA. Data are presented as mean ± S.E.M. from 4 experiments. One way ANOVA/Bonferroni *P<0.05, **P<0.01.
